# Formulation and *In Vitro* Evaluation of Furosemide Floating Matrix Tablets Using *Boswellia papyrifera* Resin as Matrix Forming Polymer

**DOI:** 10.1155/2023/4322375

**Published:** 2023-10-26

**Authors:** Adane Yehualaw, Chernet Tafere, Zewdu Yilma, Solomon Abrha

**Affiliations:** ^1^Department of Pharmacy, College of Medicine and Health Sciences, Bahir Dar University, Bahir Dar, Ethiopia; ^2^Department of Pharmaceutics, School of Pharmacy, Mekelle University, Mekelle, Ethiopia

## Abstract

The stomach and upper part of the small intestine are where furosemide is primarily absorbed when treating edema brought on by congestive heart failure (CHF), hepatic cirrhosis, renal impairment, and nephrotic syndrome. This narrow absorption window is responsible for furosemide's limited oral bioavailability. So creating a gastroretentive floating tablet could be beneficial. Natural polymers are advised for use in medication delivery because they are readily available in nature, biodegradable, relatively inexpensive, biocompatible, and nontoxic. Olibanum is a natural plant-based polymer obtained from Boswellia genus of trees and mainly composed of alcohol-soluble resin (65-85%). Ethiopia is rich in Boswellia species, with Boswellia papyrifera being the most important oleo-gum resin-producing tree species. In order to formulate a gastroretentive floating matrix tablet of furosemide, this study looked at the use of naturally occurring, locally available B. papyrifera resin as a matrix-forming polymer. By directly compressing B. papyrifera resin and HPMC K4M as matrix-forming polymers and sodium bicarbonate as a gas-generating agent, floating matrix tablets were created. The effects of the formulation variables polymer type, polymer concentration, polymer ratio, and percentage of the floating agent on the floating lag time, total floating time (duration), and cumulative drug release in 12 hours were investigated. Furosemide floating tablets formulated employing higher (40%) polymer concentrations (olibanum resin, HPMC K4M, or in combination) and 10% sodium bicarbonate as gas-generating agent demonstrated a floating lag time of less than 6 minutes and a sustained release with a total floating time of more than 12 hours. Comparing the release characteristics of floating tablets made with 40% of a total polymer and 10% of NaHCO3 revealed that the medicine is released very slowly when polymers were combined. The matrix-forming polymer, olibanum resin, obtained from *B. papyrifera*, was effectively used to make furosemide floating matrix tablets. The olibanum resin from *B. papyrifera* can therefore be used as a potential substitute matrix-forming polymer in the production of effervescent floating matrix tablets.

## 1. Introduction

Among the several drug administration methods, the oral route is the most practical and optimal since it is patient-compliant, easy to administer, has the fewest sterility restrictions, and has variable dosage form design [1]. Solid dosage forms, such as tablets and capsules, are the primary options among the variety of oral devices for the majority of active pharmaceutical ingredients (APIs), as they are simple to prepare, conveniently stored, transport, frequently more stable than their liquid counterparts, and exhibit a high level of patient acceptability [2].

However, there are issues with the traditional oral route, including the inability to restrain and localize the drug delivery system within desired regions of the gastrointestinal tract (GIT), a variable and unpredictable gastric emptying rate, a quick gastrointestinal transit time, and the existence of an absorption window for some drugs [1]. Short gastric residence time and unpredictably high gastric emptying rates have been seen in scintigraphic experiments, which have affected even controlled release formulations intended to release the drug slowly into the gastrointestinal tract (GIT) and maintain an effective drug concentration in the systemic circulation for a long time [3]. Drugs may exhibit region-specific absorption (low absorption window throughout the GIT) as a result of varying drug solubility and stability in different parts of the GIT brought on by differences in ambient pH and the presence of various enzymes [4]. The development of sustained/controlled release medications is significantly hampered by the absorption window, which restricts the bioavailability of pharmaceuticals taken orally. Drugs delivered by sustained/controlled release systems waste away with negligible absorption once the absorption window has been passed due to the short transit times of dose forms and narrow absorption window [5]. Therefore, creating sustained release formulations that stay at the absorption site for a long time would be advantageous. Controlling the formulation's gastric retention time (GRT) is one of the practical methods for achieving a longer and predictable drug delivery profile in the gastrointestinal tract (GIT) [4].

Gastroretentive dosage forms (GRDF), or dosage forms with prolonged GRT, introduce novel and significant treatment possibilities, particularly useful for sparingly soluble and insoluble medicines. The time available for drug dissolution is insufficient for drugs with lower solubility, and as a result, transit time has a substantial impact on drug absorption [6]. Delayed gastric emptying helps poorly soluble pharmaceuticals dissolve and is beneficial for medications that are mostly absorbed from the stomach or proximal region of the intestine [7]. By using the mechanisms of flotation, mucoadhesion, sedimentation, expansion, changed shape systems, or the concurrent administration of pharmacological drugs that delay gastric emptying, solid dosage forms can be retained in the stomach under controlled conditions [3, 8].

The treatment of edema brought on by congestive heart failure (CHF), hepatic cirrhosis, renal dysfunction, and nephrotic syndrome typically involves the use of furosemide, a high-ceiling loop diuretic [7]. Its low bioavailability of around 50% and extremely variable (erratic) oral bioavailability of 37-51% are caused by its limited absorption window, which is largely in the stomach and upper small intestine [9]. A reason for creating a gastroretentive effervescent floating tablet of furosemide is provided by the medication's limited absorption window in the stomach and upper GIT [7, 10]. In order to target the medication delivery at a specific location in the GI tract, such as the stomach, floating drug delivery systems use both synthetic and natural polymers [11]. Natural polymers are more widely used for making floating matrix tablets because they are readily available, biodegradable, capable of chemical modification, reasonably priced, biocompatible, and nontoxic [12]. *Boswellia papyrifera* is the main indigenous tree in Ethiopia that produces oleo-gum resin, and olibanum extracted from this plant contains 65–85% resin [13, 14]. In the food and pharmaceutical industries, olibanum is a phytotoxically safe raw ingredient [15]. This study looks at the local B. papyrifera resin's potential as a matrix-forming polymer in the creation of gastroretentive effervescent floating matrix tablets using furosemide as the active ingredient.

## 2. Materials and Methods

### 2.1. Materials

Furosemide API and reference standard were supplied by Addis Pharmaceutical Factory (APF), HPMC K4M (Dow Chemical Company) was donated by Cadila Pharmaceuticals PLC, Talc was supplied by the pharmaceutics laboratory of Mekelle University, *B. papyrifera* oleo-gum resin was purchased from the Ethiopian Natural Gum Processing and Marketing Enterprise (NGPME), and NaHCO_3_ (Abron Chemicals, India), lactose monohydrate (Loba Chemie PVT.LTD, India), magnesium stearate ethanol, HCl, and NaOH were all used as received.

### 2.2. Methods

#### 2.2.1. Preparation of Olibanum Powder

The olibanum purchased from the Ethiopian Natural Gum Processing and Marketing Enterprise (NGPME) was dried in an oven (Memmert, Germany) at 60°C for 4 hrs. The dried olibanum was powdered using maximan coffee grinder (Maximan, MAX 9255, China) and passed through a mesh having a pore size of 250 *μ*m [13].

#### 2.2.2. Isolation of Olibanum Resin from Gum Olibanum

Extraction of resin was done as per the method described in articles [16]. In order to extract the resin, the powdered olibanum oleo-gum resin was stirred with 90% ethanol for 2 hrs making solute solvent ratio 1 gm to 10 ml. The ethanol slurry was filtered through Whatman No. 1 filter paper, and the filtrate (oleo resin) was concentrated to a thick paste by evaporation of ethanol at 80°C. Then, the thick paste was hydrodistilled using distillation apparatus for 3 hrs, and essential oils were separated. The paste was finally dried in an oven (Memmert, Germany) at 40°C for about 2 weeks, and the dry mass was powdered using maximan coffee grinder (Maximan, MAX 9255, China) and passed through a mesh having a pore size of 250 *μ*m.

#### 2.2.3. Drug Excipient Compatibility Study

Drug excipient interaction was studied by Fourier transformed infrared (FT-IR) spectroscopy. FT-IR spectra for pure furosemide, pure olibanum resin, and physical mixture of all excipients for furosemide matrix tablets were acquired at room temperature using FT-IR spectrophotometer. The samples were first ground in a mortar, and finely ground powder of each sample was mixed with an oily mulling agent (Nujol) in a mortar and pestle. The sample mixture was then placed onto the face of a potassium bromide (KBr) disk, and the second window was placed on top of the first salt plate to form a thin film of the mull by compression between two plates. The sandwiched plates were placed in the spectrometer, and the spectra were obtained by scanning between wave numbers 4000-400 cm^−1^ using potassium bromide discs [17].

#### 2.2.4. Determination of UV Absorption Maximum (*λ*_max_) and Standard Calibration Curve of Furosemide

Determination of the maximum wavelength of absorption was conducted using a T80 UV/visible spectroscopy. Furosemide stock solutions of 1 mg/ml in 0.1 M NaOH (pH 13) and 0.1 mg/ml in 0.1 N HCl (pH 1.2) were prepared.

For the determination of absorption maximum of furosemide in 0.1 M NaOH, 100 mg of a pure furosemide powder was weighed and dissolved in a 100 ml volumetric flask using 0.1 M NaOH solution. The volume was made up to 100 ml using 0.1 M NaOH to obtain a 1 mg/ml preparation which was scanned in the range of 200-400 nm of the spectrophotometer [18]. The wavelength of maximum absorption (*λ*_max_) was determined to be 240 nm.

The calibration curve of reference standard furosemide in 0.1 M NaOH was generated by preparing dilutions from a stock solution of 1 mg/ml. Different concentrations of furosemide reference standard solutions were prepared to obtain 2 *μ*g/ml, 4 *μ*g/ml, 8 *μ*g/ml, 12 *μ*g/ml, 16 *μ*g/ml, and 20 *μ*g/ml in 0.1 M NaOH. Then, samples were analyzed using UV-visible spectroscopy at the determined *λ*_max__,_ and the measured absorbance values of each sample were plotted versus concentration to obtain the standard calibration curve [18].

For the determination of absorption maximum of furosemide in 0.1 N HCl, 25 mg of a pure furosemide powder was weighed and dissolved in a 250 ml volumetric flask using 0.1 N HCl solution. The volume was made up to 250 ml using 0.1 N HCl, and from this stock solution of 0.1 mg/ml prepared, 10 ml was taken and diluted to 100 ml to obtain 1 mg/100 ml solution. The 1 mg/100 ml solution was scanned by UV-visible spectrophotometer at the range of 200-400 nm, and the absorption maximum (*λ*_max_) of the drug was determined [7] to be 234.75 nm.

Then, different concentrations of furosemide reference standard solutions were prepared from a stock solution of 10 mg/100 ml to obtain 2 *μ*g/ml, 4 *μ*g/ml, 6 *μ*g/ml, 8 *μ*g/ml, 10 *μ*g/ml, 12 *μ*g/ml, 14 *μ*g/ml, 16 *μ*g/ml, 18 *μ*g/ml, and 20 *μ*g/ml in 0.1 N HCl. Then, samples were analyzed spectrophotometrically at the determined *λ*_max_. The measured absorbance values of each sample were plotted versus concentration, and a standard calibration curve was generated [7].

#### 2.2.5. Preparation of Powder Blend for Compression

The powder blend to be directly compressed was prepared according to the design depicted in Table 1. For each formulation, accurately weighed quantity of furosemide (API), respective polymer (either olibanum resin or HPMC K4M or both), lactose, and sodium bicarbonate were manually blended to get a homogenous blend using a pestle and mortar. The blended powder was passed through sieve #45 (355 *μ*m) and was collected in a plastic polyethylene bag and mixed for 20 minutes. To this mixed blend, talc and magnesium stearate which passed through sieve #45 (355 *μ*m) were added, and the whole mixture was mixed for 5 minutes in a plastic polyethylene bag.

#### 2.2.6. Characterization of Powder Blend

The powder blend for the formulation of matrix tablets was evaluated with respect to the following flow properties.

*(1) Angle of Repose*. The angle of repose was determined as described in literature [19] using the funnel method (made up of transparent paper) with an internal diameter of 10 mm at the bottom and 100 mm at the top. 30 gm of powder mixture was allowed to pass through the funnel fixed to a stand at a fixed height of 10 cm. The angle of repose was then obtained by using the height and radius of the heap of the powder formed. The radius of the heap (*r*) was measured, and the angle of repose (*θ*) was calculated using the formula

(1) $$\begin{array}{c} \theta =\tan^{-1}\left(\frac{h}{r}\right). \end{array}$$

*(2) Density and Density-Related Properties*. Powder blend characteristics such as bulk density, tapped density, compressibility index, and Hausner's ratio were determined as described elsewhere [19–21].

*Bulk density*: apparent bulk density (*ρb*) was determined by pouring 30 gm of powder blend into a 250 ml graduated cylinder. The bulk volume (*V*_*b*_) and weight of the powder (*M*) were determined, and the bulk density (*ρb*) was calculated using the formula *ρb* = *M*/*V*_*b*_.

*Tapped density*: the measuring cylinder containing a known mass (30 gm) of powder blend was tapped until no apparent volume change was observed. The minimum volume (*V*_*t*_) occupied in the cylinder and the weight of the blend were measured. The tapped density (*ρ*_*t*_) was calculated using the formula *ρt* = *M*/*V*_*t*_.

*Compressibility index (CI)*: the compressibility index (CI) was calculated from bulk and tapped densities: CI = *ρ*_*t*_ − *ρ*_*b*_/*ρ*_*t*_∗100.

*Hausner's ratio (H)*: it was calculated from bulk and tapped densities by the formula *H* = *ρ*_*t*_/*ρ*_*b*_.

#### 2.2.7. Formulation of Floating Tablet of Furosemide

Floating tablets were prepared by direct compression method according to the design depicted in Table 1 by using *B. papyrifera* resin and/or HPMC K4M as matrix-forming polymers. The powder blend prepared was directly compressed into tablet on a tablet minipress machine (MII Riva S. A, Argentina) by adjusting the average tablet weight to 280 mg at a constant compression force.

#### 2.2.8. Characterization of Tablets

Tablet characteristics like uniformity of weight, hardness, friability, and thickness were evaluated employing the methods described in literature [16, 19–21].

*(1) Uniformity of Weight*. 20 tablets were selected from each batch at random and weighed singly and later collectively using a digital analytical balance (Adventurer™, China). The mean weight and standard deviation were determined.

*(2) Hardness*. The hardness of 10 tablets from each batch was determined using a hardness tester (CALIVA, THT2, England), and the average value was obtained.

*(3) Friability*. Tablet friability was determined using a friability tester (ERWEKA, TAR 20, Germany) by placing 10 tablets and rotating them for 4 min at 25 rpm. Tablets were dedusted using a soft muslin cloth and reweighed, and then, the loss of tablet weight was calculated as a percentage of the initial weight after dedusting of the tablets.

(2) $$\begin{array}{c} \%\text{loss}=\frac{W_{i}-W_{f}}{W_{i}}, \end{array}$$

where *W*_*i*_ is the initial weight and *W*_*f*_ is the final weight after dedusting of tablets.

*(4) Thickness*. The thickness of 10 tablets from each batch was measured by the hardness tester (CALIVA, THT2, England) putting the tablet with their side (in an upright position), and the average value was obtained.

*(5) Evaluation of Floating Properties*. Prepared floating tablets of furosemide were evaluated for floating lag time and total floating time. The time the tablets took to emerge on the fluid surface (floating lag time) and the time the tablets constantly float on the fluid surface (floating duration) in a USP type II apparatus containing 900 ml of 0.1 N HCl solution, maintained at 37 ± 0.5°C with stirring speed of 50 rpm, were recorded using stopwatch [8].

*(6) Matrix Integrity*. Matrix integrity of the swollen mass of tablets was checked through visual observation throughout the *in vitro* dissolution study [4].

*(7) Drug Content Determination (Assay)*. Twenty tablets were randomly selected from a batch of furosemide tablets and finely powdered. A quantity of powder equivalent to 0.2 gm of furosemide was transferred to 500 ml volumetric flask and shaked with 300 ml of 0.1 M NaOH for 10 minutes. Sufficient of 0.1 M NaOH was added to produce 500 ml and filtered. 5 ml of the above solution was diluted into 250 ml 0.1 M NaOH, and the absorbance of the resulting solution was measured at the wavelength of maximum absorption (*λ*_max_) determined [20].

*(8) In Vitro Drug Release Studies*. *In vitro* release of furosemide floating tablet formulations was determined using USP type II apparatus (paddle method) (PHARMA TEST, Germany) for a period of 12 hrs. Samples of tablets for each formula were evaluated for *in vitro* drug release using a dissolution medium of 900 ml in 0.1 N HCl, pH 1.2 at 37.0°C ± 0.5 with stirring speed of 50 rpm. Aliquot samples of 10 ml were withdrawn at prescheduled intervals (0.5, 1, 2, 4, 6, 8, 10, and 12 hr) and replaced with an equal volume of fresh dissolution medium which was kept at 37 ± 0.5°C to maintain sink condition. Then, it was filtered (membrane pore filter 0.45 *μ*m) and diluted suitably and analyzed for the drug content at the scanned *λ*_max_ (234.75 nm) using a UV/visible spectrophotometer [8, 17, 22]

*(9) Drug Release Kinetics and Mechanism*. The dissolution data were fitted into different equations of drug release kinetic models to evaluate the rate and mechanism [10, 23] of furosemide release from prepared floating matrix tablets. The release profile obtained from each batch was fitted to zero order, first order, the Higuchi square root model, the Korsmeyer–Peppas model, and the Hixon-Crowell cube root models to determine the release kinetic and drug release mechanism.

*Zero-order equation*: *Q* = *Q*_0_ + *K*_0_*t*, where *Q* is the amount of drug released, *Q*_0_ is the initial amount of drug in solution (it is usually zero), and *K*_0_ is the zero-order release constant. The plot was made as cumulative % drug release vs. time.

*First-order equation*: ln*Q* = ln*Q*_0_–*K*_1_*t*, where *Q* is the amount of drug remaining at time *t*, *Q*_*o*_ is the quantity of drug present initially in the dosage form, and *K*_1_ is the first-order release constant. The plot was made as log cumulative % drug remaining vs. time.

*Higuchi's square root model*: Higuchi derives an equation to describe the release of a drug from an insoluble matrix as the square root of a time-dependent process [24], *Q* = *Kt*^1/2^, where *Q* is the amount of drug released at time *t* and *K* is the constant reflecting the design variables of the system. The plot was made as cumulative % drug release vs. square root of time.

*Hixson-Crowell cube root model*: *Q*_*t*_^1/3^ = *Q*_0_^1/3^ − *K*_HC_*t*, where *Q*_*t*_ is the amount of drug remaining at time *t*, *Q*_0_ is the initial amount of drug in the tablet, and *K*_HC_ is the rate constant for the Hixson-Crowell cube root model. The plot was made as a cube root of drug % remaining in matrix vs. time.

*Korsmeyer–Peppas model*: To find out the mechanism of drug release, first 60% drug release data was fitted in Korsmeyer–Peppas model, *Q*_*t*_/*Q*_0_ = *Kt*^*n*^, where *Q*_*t*_ is the amount of drug released at time *t*, *Q*_*o*_ is the amount of total drug in tablets, *Q*_*t*_/*Q*_*o*_ is the fractional drug release at time *t*, *K* is a constant incorporating the structural and geometric characteristics of the matrix tablets, and *n* is a diffusional exponent, indicative of the drug release mechanism. The plot was made as log cumulative % drug release vs. log time, and the slope *n* (release exponent) was determined. The release mechanisms for cylindrical-shaped matrices are given in Table 2.

#### 2.2.9. Statistical Analysis

The statistical analysis of data was performed with Microsoft Excel and Origin 8 Software (Origin Lab Corporation, MA and USA). One-way analysis of variance (ANOVA) was used for comparison of results.

## 3. Results and Discussion

### 3.1. Drug Excipient Compatibility Study

Drug excipient interaction was studied by Fourier transformed infrared (FT-IR) spectroscopy. FT-IR spectra for pure furosemide, pure olibanum resin, and physical mixture of excipients for furosemide matrix tablets were acquired at room temperature using FT-IR spectrophotometer.

From the infrared spectrum of pure furosemide, absorption bands were observed at 3395, 3347, 3254, 2953, 1668, and 1560 cm^−1^ which are the functional group regions of the pure drug spectrum [25]. The 3395 cm^−1^ band is assigned to the NH_2_ stretching vibration of Ar-NHCH_2_ (secondary amine), 3347 and 3254 cm^−1^ band is assigned to the stretching vibration of SO_2_NH_2_ primary amines, and the broad band observed at 2953 cm^−1^ is assigned to the acidic OH group. The sharp bands observed at 1668 cm^−1^ band are assigned to the bending vibration of the amino group, and the 1560 cm^−1^ band is due to the asymmetric stretching vibration of the carbonyl group [17, 25]. These characteristic peaks also appear in the spectrum of the floating furosemide tablet formulations at the same wave numbers indicating that there was no interaction between the drug and formulation excipients.

### 3.2. Determination of Absorption Maximum (*λ*_max_) and Calibration Curves of Furosemide

**Figure 1:**
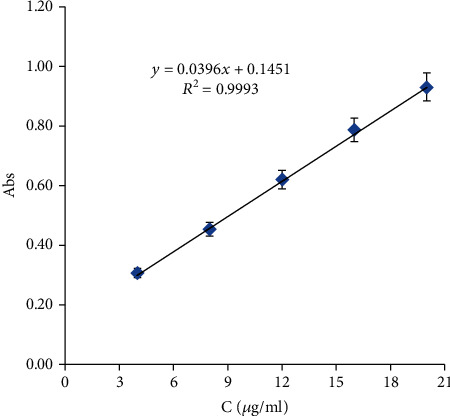
Calibration curve of reference standard furosemide dissolved and diluted with 0.1 N HCl.

Calibration curves of reference standard furosemide were generated using 0.1 N HCl and 0.1 M NaOH as dilution medium. The absorbance reading of each of the different concentrations of furosemide reference standard solutions prepared were measured at the scanned *λ*_max_ of 234.75 nm using T80 UV/visible spectroscopy. The calibration curve of furosemide in 0.1 N HCl showed linearity in a concentration range of 4 to 20 *μ*g/ml with a determination coefficient (*R*^2^) of 0.9993 with an equation of *Y* = 0.0396 × +0.1451 (Figure 1).

**Figure 2:**
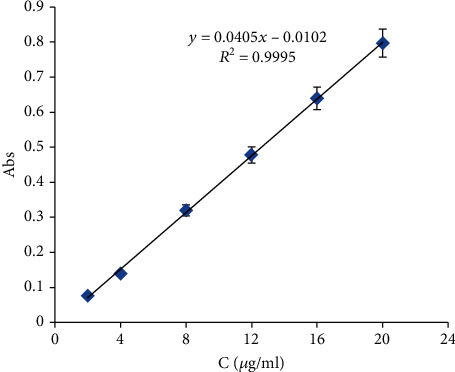
Calibration curve of reference standard furosemide dissolved and diluted with 0.1 M NaOH.

The calibration curve of reference standard furosemide in 0.1 M NaOH (Figure 2) was generated by preparing dilutions from a stock solution of 1 mg/ml. Samples were analyzed spectrophotometrically at the determined *λ*_max_ of 240 nm, and the measured absorbance values of each sample were plotted versus concentration to obtain the standard calibration curve. The generated calibration curve of furosemide in 0.1 M NaOH showed linearity in a concentration range of 2 to 20 *μ*g/ml with a determination coefficient (*R*_2_) of 0.9995 and with an equation of *Y* = 0.0405 × −0.0102.

### 3.3. Formulation Studies

The olibanum resin used for the formulation of floating tablets of furosemide was obtained from *B. papyrifera* species. The color of the resin powder obtained by alcoholic extraction of an oleo-gum resin of *B. papyrifera* was light yellow. The percentage yield of resin fraction was found to be 67.3% (w/w) which is in good agreement with the reported range of 65-85% in the literature [16, 26].

Floating matrix tablets of furosemide were prepared employing olibanum resin, a natural resin of *B. papyrifera*, HPMC K4M, and combination of the two with an objective of evaluating the resin as matrix former in effervescent floating tablets. The floating matrix tablets of the resin were comparatively evaluated with floating matrix tablets of HPMC K4M, a well-known matrix former employing sodium bicarbonate as a gas-generating (floating) agent.

HPMC could be used as drug sustaining matrix agent from 10% to 80% [27] and olibanum resin from 2% to 10% [28] and 32% to 57% [16]. The low and high levels of the matrix-forming polymers used in the current study were 10% and 40%. Sodium bicarbonate is reported to be used as a floating agent from 10% to 20% [4, 29].

#### 3.3.1. Powder Blend and Tablet Characteristics

As shown in Table 3, all formulations exhibited good to fair flow characteristics. Formulations have an angle of repose ranging from 26.3° to 32.34°, which indicates an excellent to good flow property of the powder. Hausner's ratio ranged from 1.13 to 1.21, and the compressibility index ranged from 11.33 to 17.48. The results of compressibility index and Hausner's ratio support for the good to fair flow properties of the powder blend. The Carr's index and Hausner's ratio of those formulations with higher proportion of olibanum resin showed relatively good flow properties which is in agreement with Kebebe et al. [16], finding that Carr's index and Hausner's ratio of the resins showed good flow properties of the powder. Those formulations with only HPMC and higher proportion of HPMC (in the case of the combination formulations) showed fair to good flow property.

Tablets were prepared by direct compression method due to the ease and simplicity of the method [8, 30], and direct compression can be used frequently for tablets containing 25% or less of drug substances with a suitable diluent that acts as a carrier or vehicle for the drug [31].

The formulated floating matrix tablets were evaluated for uniformity of weight, hardness, friability, thickness, and drug content assay (Table 4). The average weight of the tablets was found within the pharmacopeial limit (±5% of average weight) [20]. In order to withstand mechanical shocks of handling during its manufacture, packaging, transport, and reasonable abuse on the hands of the consumer, the tablet requires a certain amount of strength or hardness [6]. As shown in Table 4, the thickness of the tablets ranged between 2.58 to 3.63 mm, and the hardness of the tablets was between 4.48 kg/cm^2^ and 6.19 kg/cm^2^. The friability was found to be less than 1% for all formulations except B_11_, which is an indication of satisfactory mechanical strength of the tablets. Friability greater than 1% of formulation B_11_ might be due to the higher percentage of sodium bicarbonate which is a material with poor bonding properties [32] and the higher percentage of olibanum resin which has brittle nature [16]. The drug content analysis showed values in the range of 96.76 to 102.62% reflecting good uniformity among different formulations [20, 21]. All the formulations that contain low level of polymer concentration (10%) could not keep their matrix integrity for 12 hr. Except B_11_, all formulations that contain high level of polymer concentration (40%) keep their matrix integrity for more than 12 hr. The rapid matrix rapture of B_11_ may be associated with an increase in rate of pore formation of the tablets due to an increase in the percentage of effervescent agent [33]. All the formulations showed satisfactory values of hardness, friability, and drug content indicating that the prepared tablets were of standard quality.

#### 3.3.2. Comparison of Formulations

As release-sustaining matrix forming agents, olibanum resin, HPMC K4M, and a mixture of the two were used in the formulation of floating matrix tablets. Floating lag time, total floating duration, and cumulative drug release over 12 hours were taken into consideration while comparing these formulations [4, 8].

As depicted in Table 5, all batch of formulations floated after a lag time of less than 6 minutes which is shorter than the time in which motor activity in the fed state is induced.

As shown in Table 5 above, the formulations containing lower concentrations of polymers (either alone or in combination) could not sustain the drug release for long, and their matrix disintegrates shortly. Formulations with only 10% of olibanum resin (B_1_) floated for 2 hrs and release 70.25% of their content within 1 hr, which is an indicative of the chance of dose dumping [19]. The one with 10% of HPMC only and two of the formulations at 10% of total polymer (resin/HPMC; 1 : 1 and 1 : 3) disintegrated within minutes of the dissolution study. The one with resin/HPMC ratio of 3 : 1 (at 10% of total polymer) showed a burst release of 78.7% at the first 1 hr even though it could float for about 1.8 hr. This may be due to the release of drug particles present on the surface of the matrix system into the surrounding media generating many pores and cracks which facilitate further release of drug with the formation of channels within the matrix due to lower concentration of polymer that could not entrap the drug and the effervescent agent which provides pore for faster release of the drug [4]. Therefore, the rapid drug release from the tablet was due to the reduced amount of polymer that could not control the drug release.

Formulations containing 40% of total polymer concentration either alone or in combination were able to float and sustain the drug release for more than 12 hrs with the exception of B_11_. The formulations with 40% of resin alone floated for more than 12 hrs at lower level of sodium bicarbonate (10%), and the cumulative release was 98.5% in 12 hrs. But at higher level of NaHCO_3_ (20%), it did not float for more than 3.8 hrs and experiences a burst release of 62% within 1 hr. This may be attributed to an increase in rate of pore formation and rapid hydration of the tablets due to an increase in the percentage of effervescent agent [33], and an increase in content of the gas-generating agent increases tablet float but quickly disintegrates [8].

Formulations with HPMC alone sustained the drug release for 12 hrs and were able to float for more than 12 hrs at 40% concentration, and the cumulative release was found to be 97.25% and 99.00% in 12 hrs. There was not significant change (*p* > 0.05) in floating lag time, cumulative release in 12 hr, and total floating time when NaHCO_3_ level changed in this type of formulations (keeping HPMC K4M concentration 40%).

Formulations with higher polymer concentrations of combination of resin and HPMC showed a good sustained release pattern. Comparison of release profiles of floating tablets prepared with the combination of polymers and two polymers alone indicated that the drug release was relatively slow in case of combination of polymers. Those formulations at 40% of the total polymer (olibanum resin/HPMC; 1 : 1, 1 : 3, and 3 : 1) floated for more than 12 hrs and sustained the drug release for more than 12 hrs. The cumulative release in 12 hrs was 80.62, 68.6, and 80.32%, respectively. These formulations float after a lag time of below 10 seconds. This is due to the importance of combining polymers in order to improve weakness of one polymer with the other polymer. HPMC has a short floating lag time compared to olibanum resin which shows longer floating lag time (delayed floating) [29]. The rapid floatation within seconds of the dissolution study of HPMC containing formulations and the lag in floating time of only olibanum resin containing formulations may be associated with the rapid hydration due to hydrophilic nature of HPMC and less hydrophilic nature of the resin, respectively. In comparison of release profiles of floating tablets prepared with the two polymers alone indicated that the drug release was relatively rapid with olibanum in the initial phase. This might be associated with the rapid hydration and swelling so that could prevent release of drug due to hydrophilic nature of HPMC and less hydrophilic nature of the resin. These findings were in agreement with the results of the study on combination of olibanum gum resin with synthetic hydrophilic polymer and resulted promising properties for gastroretentive floating drug delivery to maintain the dimensional stability at initial stage [34].

In general furosemide floating tablets formulated employing higher percentage of olibanum resin, HPMC K4M and in combination as matrix formers and sodium bicarbonate as gas-generating agent exhibited a floating time of more than 12 hours. Furosemide release from the floating tablets prepared was mainly dependent on the polymer and sodium bicarbonate concentrations used. Formulations with higher polymer concentrations of combination of resin and HPMC K_4_M showed a good sustained release pattern with dimensional stability. Comparison of release profiles of floating tablets prepared with the combination of polymers and two polymers alone indicated that the drug release was relatively slow in the case of combination of polymers. This may be due to an inter- and intra-cross-linking of combination of the two polymers that resulted a strong tortuosity to the matrix formed [35]. Olibanum resin was found to be suitable matrix forming polymer in the formulation of floating matrix tablets especially when it is combined with the synthetic polymer HPMC K_4_M according to the finding of our study. Combining minimizes the brittleness of the resin matrix. Therefore, formulation B_10_, which is the 3 : 1 ratio of resin and HPMC K_4_M, would be the best formulation we suggest.

### 3.4. *In Vitro* Drug Release

**Figure 3:**
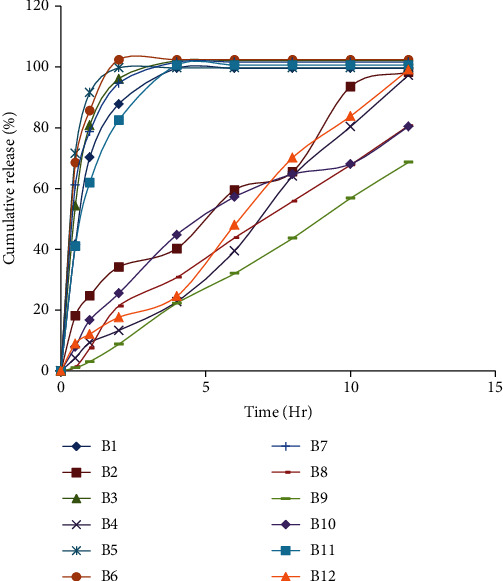
The *in vitro* drug release profile of furosemide formulations.

Using USP type II equipment (paddle method), the *in vitro* release of furosemide from floating tablet formulations was assessed over the course of 12 hours. The concentration of the polymer and the concentration of NaHCO3 seem to have a significant impact on the drug release pattern, as seen by the drug release profiles of the various formulations (Figure 3).

### 3.5. Drug Release Kinetics

A linear regression analysis of the *in vitro* drug release data can be used as an indicator of the release kinetics model and mechanism from matrix delivery systems. Dissolution data were fitted and evaluated using zero order, first order, the Higuchi square root, and the Hixson-Crowell cube root model kinetic equations to understand the release kinetics. This was done for formulations that could sustain the release for long (B_2_, B_4_, B_8_, B_9_, B_10_, and B_12_). Because of their initial burst release of greater than 30% within 1 hr, the rest were not evaluated. The results shown in Table 6 present the linear regression results of the model fitting tests of the formulations. Coefficients of determination (*R*^2^) were used to evaluate the goodness of the model fit. As shown in Table 6, the Higuchi square root model showed the best fit with high linearity for formulation batch's B_2_ (*R*^2^ = 0.85), B_4_ (*R*^2^ = 0.96), B_8_ (*R*^2^ = 0.99), and B_10_ (*R*^2^ = 0.99). For formulation batch B_9_, the Hixson-Crowell kinetics model showed the best fit (*R*^2^ = 0.98), and for formulation B_12_, zero-order kinetics model showed the best fit (*R*^2^ = 0.99).

To evaluate the mechanism of drug release from the dosage form, *in vitro* drug release data up to the first 60% were fitted to the Korsmeyer–Peppas model, and the exponent (**n**) was calculated from the slope of the straight line log cumulative percentage of drug released vs. log time [36]. In this model, the value of **n** illustrates the type of release mechanism [10]. As depicted in Table 7, the **n** value for the formulation B_2_ was found to be <0.45 which indicates that drug release involved was mainly the Fickian diffusion. For formulations B_4_, B_10_, and B_12_, the value of **n** was found in a range of between 0.45 and 0.89 which indicates that the drug release from these formulations followed non-Fickian (anomalous) diffusion while the **n** values for B_8_ and B_9_ were found to be greater than 0.89 indicating that super case II transport was their mechanism of release. Anomalous diffusion drug release mechanism signifies a coupling of both diffusion and erosion mechanisms which indicate that the drug release is controlled by more than one process during the entire period of drug release [37, 38]. Super case II transport indicates or refers drug release that is erosion-controlled. Case II generally refers to erosion of polymeric chain [37].

## 4. Conclusion

The results of this study demonstrate that furosemide floating matrix tablets could be prepared employing olibanum resin derived from *B. papyrifera* as a matrix-forming polymer by direct compression method. It was found that the formulation variables polymer concentration, polymer type, polymer ratio, and the sodium bicarbonate concentration affected the properties of the manufactured floating tablets. Formulation of furosemide floating tablets formulated using higher polymer concentrations (olibanum resin alone, HPMC K4M alone, and the two combined) as matrix formers and sodium bicarbonate as a gas-generating agent demonstrated a floating time of more than 12 hours with good sustained release pattern. Those formulations that just contained olibanum resin displayed a considerable lag to float than formulations that also contained HPMC. The drug release was somewhat slow when the two polymers were combined, according to a comparison of the release profiles of floating tablets. Drug release from formulation B_10_, which is the 3 : 1 ratio of resin and HPMC K_4_M, was so slow (sustained) and has a matrix with dimensional stability. It would be suggested as the best formulation batch formulated. It was demonstrated that the drug release for the examined furosemide formulations was regulated by both erosion and diffusion. So, according to the findings of this study, olibanum resin obtained from *B. papyrifera* can be employed as a substitute pharmaceutical excipient in the creation of floating matrix tablets.

## Figures and Tables

**Table 1:** Composition of formulated tablets.

Form. code	Ingredients (% w/w)						
	API	Resin	HPMC K4M	NaHCO3	Lactose	Talc	Mg-stearate
B_1_	14.3	10	—	10	63.7	1	1
B_2_	14.3	40	—	10	33.7	1	1
B_3_	14.3	—	10	10	63.7	1	1
B_4_	14.3	—	40	10	33.7	1	1
B_5_	14.3	5	5	10	63.7	1	1
B_6_	14.3	2.5	7.5	10	63.7	1	1
B_7_	14.3	7.5	2.5	10	63.7	1	1
B_8_	14.3	20	20	10	33.7	1	1
B_9_	14.3	10	30	10	33.7	1	1
B_10_	14.3	30	10	10	33.7	1	1
B_11_	14.3	40	—	20	23.7	1	1
B_12_	14.3	—	40	20	23.7	1	1

**Table 2:** Diffusion exponent and release mechanism for cylindrical shape matrices [10].

Diffusion exponent (*n*)	Release mechanism
≤0.45	Fickian diffusion
0.45 < *n* < 0.89	Anomalous (non-Fickian) transport
0.89	Case II transport
*n* > 0.89	Super case II transport

**Table 3:** Powder blend properties for the formulations.

Formulation code	Bulk density (g/ml)	Tapped density (g/ml)	Compressibility index (CI)	Hausner's ratio (HR)	Angle of repose (°)
B_1_	0.61 ± 0.02	0.71 ± 0.01	14.03 ± 2.32	1.16 ± 0.03	26.92 ± 1.01
B_2_	0.61 ± 0.02	0.72 ± 0.01	14.78 ± 3.30	1.17 ± 0.04	28.33 ± 2.04
B_3_	0.60 ± 0.02	0.71 ± 0.01	15.08 ± 3.45	1.18 ± 0.04	29.1 ± 0.85
B_4_	0.61 ± 0.02	0.74 ± 0.02	17.48 ± 3.44	1.21 ± 0.05	31.2 ± 1.45
B_5_	0.57 ± 0.02	0.66 ± 0.01	14.59 ± 2.14	1.17 ± 0.03	26.3 ± 1.23
B_6_	0.59 ± 0.02	0.69 ± 0.01	14.02 ± 1.01	1.16 ± 0.01	28.1 ± 1.46
B_7_	0.63 ± 0.02	0.74 ± 0.02	14.01 ± 1.34	1.16 ± 0.02	27.6 ± 2.11
B_8_	0.58 ± 0.01	0.67 ± 0.01	13.00 ± 0.78	1.15 ± 0.01	29.4 ± 1.22
B_9_	0.57 ± 0.01	0.67 ± 0.02	15.38 ± 1.58	1.18 ± 0.04	29.8 ± 2.43
B_10_	0.61 ± 0.02	0.7 ± 0.01	12.44 ± 2.76	1.14 ± 0.03	28.4 ± 2.11
B_11_	0.60 ± 0.01	0.68 ± 0.01	11.33 ± 0.91	1.13 ± 0.02	27.4 ± 1.80
B_12_	0.59 ± 0.02	0.72 ± 0.03	17.16 ± 2.31	1.21 ± 0.03	32.34 ± 2.41

**Table 4:** Furosemide floating tablet properties.

Form. code	Mean weight (mg) ± S.D	Hardness (kg/cm2) ± S.D	Thickness (mm) ± S.D	Friability (%)	Matrix integrity 12 hr	Assay (%)
B_1_	277.6 ± 5.87	4.60 ± 0.63	2.58 ± 0.09	0.88	Nonintact	99.43
B_2_	284.55 ± 4.07	5.75 ± 0.68	2.63 ± 0.07	0.97	Intact	98.88
B_3_	280.8 ± 6.52	4.50 ± 0.52	2.60 ± 0.07	0.81	Nonintact	101.72
B_4_	272.2 ± 6.68	4.86 ± 0.51	2.56 ± 0.07	0.74	Intact	98.42
B_5_	280.6 ± 6.72	4.64 ± 0.49	2.59 ± 0.09	0.84	Nonintact	100.61
B_6_	280.4 ± 5.15	4.48 ± 0.56	2.59 ± 0.03	0.87	Nonintact	101.13
B_7_	279.8 ± 4.00	6.19 ± 0.41	2.59 ± 0.05	0.89	Nonintact	102.62
B_8_	282.95 ± 4.44	5.13 ± 0.47	2.61 ± 0.05	0.72	Intact	96.76
B_9_	275.70 ± 3.71	5.34 ± 0.34	2.58 ± 0.04	0.75	Intact	97.54
B_10_	283.10 ± 5.18	5.18 ± 0.33	2.62 ± 0.05	0.77	Intact	101.52
B_11_	285.00 ± 5.10	5.30 ± 0.41	2.64 ± 0.06	1.03	Nonintact	99.40
B_12_	273.80 ± 4.92	5.34 ± 0.37	2.57 ± 0.04	0.83	Intact	97.70

**Table 5:** Effects of formulation factors on response variables FLT, TFT, and CR in 12 hrs.

Factors	Response variables		
	FLT (sec)	TFT (hrs)	CR in 12 hrs (%)
Olibanum resin (10% NaHCO_3_)			
10% (B_1_)	36.7 ± 1.1	2	99.50
40% (B_2_)	325.2 ± 2.8	>12	98.25
HPMC K4M (10% NaHCO_3_)			
10% (B_3_)	1.6 ± 0.1	Nonintact	102.00
40% (B_4_)	3.4 ± 0.2	>12	97.25
Olibanum resin/HPMC K4M ratio (10%) (10%NaHCO_3_)			
1 : 1 (B_5_)	6.6 ± 1.6	Nonintact	99.62
1 : 3 (B_6_)	6.4 ± 1.8	Nonintact	102.32
3 : 1 (B_7_)	8.3 ± 2.3	1.8	101.55
Olibanum resin/HPMC K4M ratio (40%) (10%NaHCO_3_)			
1 : 1 (B_8_)	7.4 ± 2.2	>12	80.62
1 : 3 (B_9_)	6.8 ± 1.7	>12	68.60
3 : 1 (B_10_)	8.8 ± 2.8	>12	80.32
NaHCO_3_ (40% olibanum resin)			
10% (B_2_)	325.2 ± 3.5	>12	98.25
20% (B_11_)	315.8 ± 3.3	3.8	100.57
NaHCO_3_ (40% HPMC K4M)			
10% (B_4_)	3.4 ± 0.4	>12	97.25
20% (B_12_)	2.6 ± 0.6	>12	99.00

CR = cumulative release; FLT = floating lag time; TFT = total floating time.

**Table 6:** Rate constants and correlation coefficient fits of different kinetic equations for furosemide floating tablets.

Form. code	Zero order		First order		Higuchi square root		Hixson-Crowell	
	Slope	*R* ^2^	Slope	*R* ^2^	Slope	*R* ^2^	Slope	*R* ^2^
B_2_	6.76	0.83	-0.29	0.71	26.67	0.85	-0.25	0.76
B_4_	8.31	0.95	-0.25	0.79	33.62	0.96	-0.25	0.89
B_8_	6.95	0.98	-0.13	0.97	29.23	0.99	-0.16	0.99
B_9_	6.06	0.96	-0.10	0.97	24.998	0.96	-0.13	0.98
B_10_	5.99	0.95	-0.12	0.98	25.79	0.99	-0.15	0.98
B_12_	8.2	0.99	-0.31	0.75	33.59	0.98	-0.27	0.90

**Table 7:** Drug release mechanism of furosemide tablets evaluated by the Korsmeyer–Peppas model.

Formulation code	Exponent (*n*)	*R* ^2^
B_2_	0.28	0.99
B_4_	0.88	0.90
B_8_	2.41	0.85
B_9_	1.87	0.96
B_10_	0.75	0.99
B_12_	0.75	0.92

## Data Availability

The data used to support the findings of this study are included within the article.
